# Predicting Pharmacist Intention to Contribute to COVID-19 Management at the Community Level: A Cross-Sectional Survey Study

**DOI:** 10.3389/fpubh.2021.653335

**Published:** 2021-07-22

**Authors:** Junlei Li, Hao Hu, Wei Liu, Chi Ieong Lei, Carolina Oi Lam Ung

**Affiliations:** State Key Laboratory of Quality Research in Chinese Medicine, Institute of Chinese Medical Science, University of Macau, Macao, China

**Keywords:** pharmacist (community), COVID-19, the theory of planned behavior, intention, survey, outbreak management

## Abstract

**Introduction:** The role of pharmacists in public health management is expected to grow into a key player in the continuing measures of managing the COVID-19 pandemic, especially in the community setting. However, their intention to provide essential public health services for combating the pandemic and the impact of their attitude and beliefs are largely unknown. This study aimed to investigate the intention-to-practice COVID-19-related responsibilities of pharmacists based on the theory of planned behavior (TPB), identify the key factors predicting their intention, and explore the usefulness of the TPB model in predicting such an intention.

**Methods:** A cross-sectional, self-administered questionnaire was completed by pharmacists in Macao between May and August 2020. Quantitative responses regarding intention-to-practice COVID-19-related duties, and the four TPB variables [attitude (A), subjective norms (SN), perceived behavioral control (PBC), and past behavior (PB)] were measured. Cronbach's alpha and composite reliability were used to determine the reliability and validity of the tool. In addition to descriptive statistics, Pearson's correlation was used to determine the strengths of the association, and multiple linear regression was used to predict the association between the intention and the four key variables.

**Results:** More than half of the pharmacists practicing in Macao completed the questionnaire (296/520) giving a response rate of 56.9%. Among them, 75% were 26–40 years old and 56% were female. The majority of the participants (91.9%) demonstrated a positive intention to contribute to the COVID-19 infection management (mean = 4.19 ± 0.51). The mean scores for A, SN, PBC, and PB were 4.06 ± 0.52, 3.71 ± 0.58, 3.76 ± 0.65, and 4.03 ± 0.54, respectively. A (β = 0.671), SN (β = 0.608), PBC (β = 0.563), and PB (β = 0.829) were all positively correlated with intention (all *P* < *0.001*). It was found that 72.5% of the variance in the intention-to-practice COVID-19-related duties could be explained by the TPB model using the four key variables with A and PB being two possible predictors.

**Conclusion:** Pharmacists showed favorable A, SN, PBC, and intention in participating in COVID-19 management in the community setting. Specific training, enhanced stakeholder communication, and improved pharmacy management are essential to increase the willingness of pharmacists to take part in the COVID-19 pandemic and other public health emergencies alike in the future.

## Introduction

Since the WHO declared the coronavirus disease (COVID-19) outbreak as a public health emergency of international concern on January 30, 2020 (1), most, if not all, countries in the world are being affected. According to the COVID-19 statistics in early November, there were 49.7 million infected cases and over 1.2 million COVID-19 cases of deaths (2). It has been speculated that the profound impact of this pandemic on human health and beyond is likely to last (3). Uncertainties remain about when massive vaccination is available and how effective it is, whether effective treatment will be developed fast enough, and whether diagnostic testing could be generalized to eradicate the virus (4). Public health measures should remain focused on the resilience that ensures that the simple yet critical preventive actions (such as individual behaviors of wearing masks, hand washing, and social distancing) and vigilant measures should be continued in order to slow down the spread of the virus in the community (5, 6).

In managing and controlling the COVID-19 pandemic, pharmacists, along with other health professionals, are called upon to deliver important public health services. According to the International Pharmaceutical Federation (FIP), pharmacists in community settings are often the first point of contact with the health system and are thus well-positioned to extend essential public health services to the general public (6). Apart from ensuring the continuity in the supply of medicines and related products and the provision of pharmaceutical care, community pharmacists also bear the responsibilities of informing and educating the public, promoting infection control and prevention, and making appropriate referral (7). Indeed, pharmacists across the countries have risen to the COVID-19 challenges, responding quickly to play a role in combating this pandemic (8–12). More and more studies have been focusing on community pharmacists and their preparedness for the COVID-19 pandemic (13), willingness to provide COVID-19 testing (14, 15), COVID-19 practices and services (16), and occupational exposure (17).

While pharmacists are expected to continuously play extended roles in public health in the community settings, COVID-19 duties are yet to be incorporated into routine pharmacy practice. The current literature already informed about the need for close attention to the psychosocial dilemma of pharmacists. For instance, a cross-country needs assessment confirmed that pharmacists were worried about the impact of COVID-19 on them personally and professionally and found it difficult to work effectively during the pandemic (18). Community pharmacists in the United Kingdom were challenged with drug shortages, disruptions in their routine practice due to increased customer traffic, and inappropriate behavior from patients and their caregivers (16). In the United States, the availability of personal protective equipment (PPE) and other infection control measures around interactions between staff and customers were causes of concern for community pharmacists to ensure continuity in their important role (13). Pharmacists in Lebanon were concerned about their lack of experience and training in managing public health emergencies (19). For the changes in pharmacist practice to occur and sustain, it is important to understand the psychosocial factors that may encourage and support pharmacists to expand their role related to the pandemic. However, the intention of pharmacists to provide essential public health services combating the pandemic and the impact of their attitude and beliefs on their intention are largely unknown.

As Dawoud et al. depicted, there is a need for research to inform pharmacy practice and supporting strategies (20). The outcome should be exerting an effect on authentic issues to benefit the public, patients, and pharmacists eventually (21). For this, adopting a theoretically informed approach is important as it provides a foundation for the researcher to gather, interpret, and analyze data in a systematic manner (22). Nevertheless, the use of theoretical frameworks to explain the perceptions and behaviors of pharmacists to provide cognitive services is not common. Among the limited examples, the organizational model was used to identify barriers and facilitators relevant to implementing clinical pharmacy service in China (23). The transtheoretical model and the attitude, social influence, and self-efficacy (ASE) model had been used to determine the psychosocial and behavioral determinants of implementing and developing pharmaceutical care in Spain (24). Overall, the theory of planned behavior (TPB) was used more widely in developing clinical pharmacy practice as explained in the following.

The TPB is one of the most widely tested theoretical frameworks explaining and predicting the intention of an individual to engage in a specific behavior within a specific context (25, 26). According to the TPB, attitudes (A), subjective norm (SN), and perceived behavioral control (PBC) are the key variables, which influence the behavior through the impact on behavioral intention. An earlier review found that, according to the results of 185 independent studies, the TPB accounted for 27 and 39% of the variance in behavior and intention, respectively (27). In pharmacy practice, TPB was purposively used to explore factors associated with the intention of pharmacists to perform a range of cognitive services, such as providing pharmaceutical care services (28), educating patients about prescription drug misuse (29), providing medication therapy management services (30), reporting serious adverse drug events to the authority (31), utilizing a prescription drug monitoring program (32), and so on. These studies had concluded that TPB could appropriately help explain the intention of pharmacists across different cognitive services.

The TPB is also flexible for the inclusion of other independent predictors if they can increase the proportion of the explained variance of intention over and above that explained by the three key factors of A, SN, and PBC (33). It has been argued that behaviors are often determined by the past behavior (PB) of an individual (34). Evidence from studies also supported that the PB frequency was another important predictor of behavioral intention and thus the actual behavior (35, 36). For instance, PB was found to contribute an extra 19% variance to the prediction of physical activity controlling for other TPB variables (37). PB had been added to the TPB model in health-related studies, which supported the inclusion of PB as a variable to improve the predictive power of the TPB (38, 39). As such, in addition to the three key variables (A, SN, and PBC), PB construct was also included in the research model.

**Figure 1:**
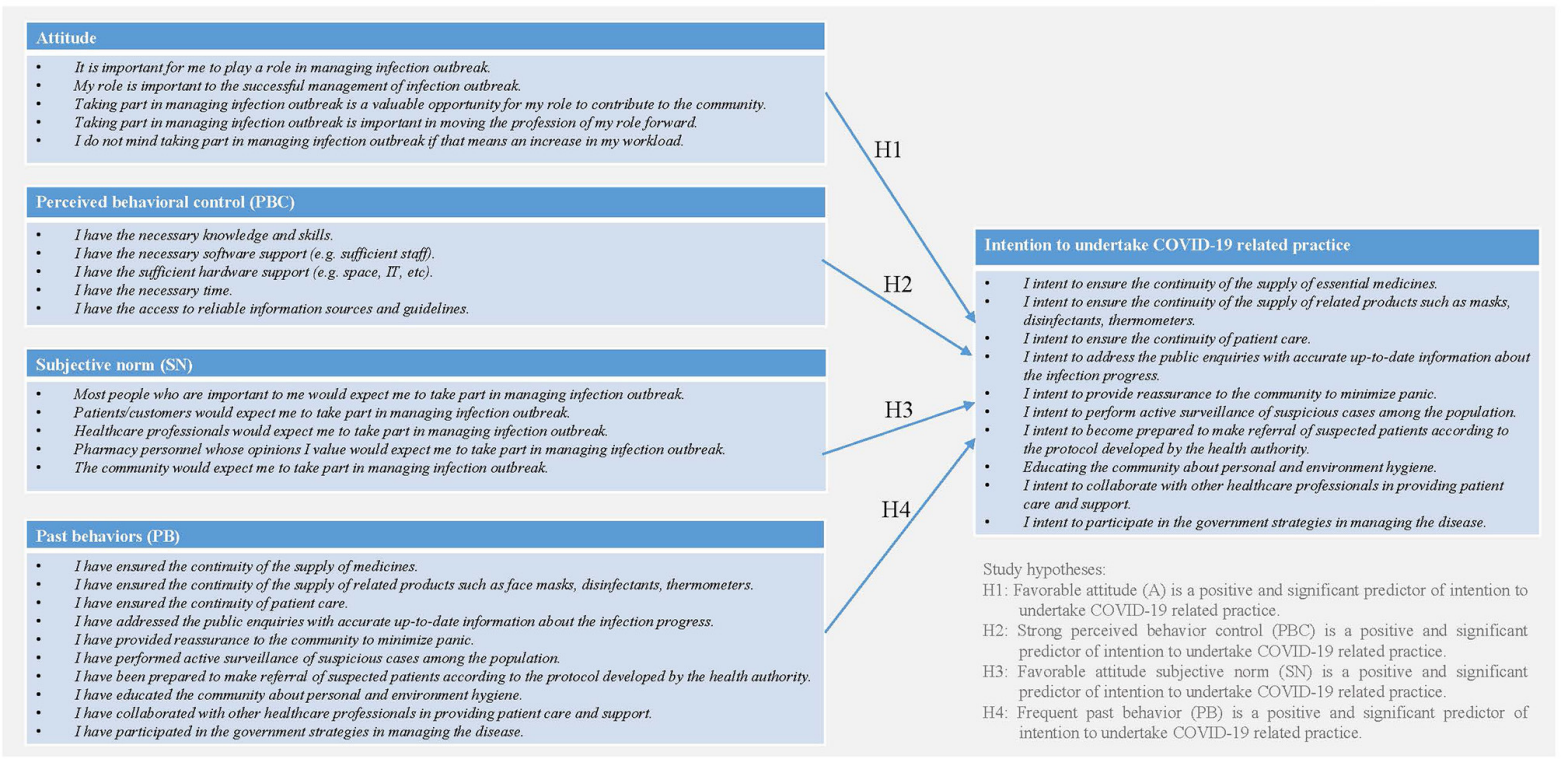
Research model of pharmacists' intension to undertake COVID-19 related practice based on the theory of planned behavior (TPB).

The behavior of interest investigated in the present study is the role of pharmacist in COVID-19-related management in a community setting. As depicted in Figure 1, regarding the four constructs in the TPB model employed in this study, A referred to the degree to which a pharmacist had a favorable or unfavorable evaluation of the COVID-19-related practice, SN referred to the belief about whether peers and people of importance to the pharmacists thought he or she should engage in the COVID-19-related practice, PBC referred to the perception of the pharmacist on the level of ease or difficulty of undertaking the COVID-19-related practice, and PB referred to his/her frequency of undertaking the COVID-19-related practice previously. The study hypotheses are, therefore, as follows:

H1: Favorable attitude (A) is a positive and significant predictor of intention-to-undertake COVID-19-related practice.
H2: Strong perceived behavior control (PBC) is a positive and significant predictor of intention-to-undertake COVID-19-related practice.
H3: Favorable attitude subjective norm (SN) is a positive and significant predictor of intention-to-undertake COVID-19-related practice.
H4: Frequent past behavior (PB) is a positive and significant predictor of intention-to-undertake COVID-19-related practice.

This study aimed to (1) investigate the intention-to-practice COVID-19-related responsibilities of pharmacists by employing the TPB model; (2) to identify the main factors predicting their intention; and (3) to explore the usefulness of the TPB model in predicting such intention. The study findings will help to inform how to improve COVID-19 management strategies and foster the public health measures through better support and integration of pharmacists in the paradigm of ongoing challenges associated with the COVID-19 pandemic.

## Methods

This study employed a cross-sectional survey informed by the TPB framework and self-administered online by pharmacists in Macao between May and August 2020. The research has been approved by the Panel on Research Ethics of the University of Macau (SSHRE20-APP029-ICMS). Following the Strengthening the Reporting of Observational Studies in Epidemiology (STROBE) guideline (40), the reporting of the study is as follows.

### Study Target

Macau is one of the most densely populated places in the world with a population of 682,800 (in the third quarter of 2020) and a famous tourist destination, exposing the city to a high risk of community transmission and imported cases amid the COVID-19 pandemic. As of early November 2020, the city had 46 cumulative confirmed cases of COVID-19 with the first case confirmed on January 22, 2020, the 46th case reported on June 26, 2020, and all of those having recovered from the disease (41). The pharmacist profession was quickly mobilized to participate in the interdisciplinary mitigation strategies led by the government. Pharmacists in the community setting, in particular, played a key role in informing and educating the public, supporting individual behavioral change in personal and environmental hygiene, being in the frontline distributing facemasks under the government arrangement, and reinforcing vigilance of suspicious cases (42). According to the official statistics, there were around 698 registered pharmacists, of whom 520 were practicing in Macao and 370 were working at 296 community pharmacies and other community settings (43). This represented 102 registered pharmacist/100,000 population and 43 community pharmacies/100,000 population ratios higher than those of the OECD average (80 pharmacists/100,000 population and 25.1 community pharmacies/100,000 population) (44). In light of the pre-existing public–private partnership between the community pharmacy and the government, and the infrastructure for data sharing (42), pharmacist service in the community setting is expected to continuously play a significant role in the evolving COVID-19 management strategy.

### Questionnaire Design

The questionnaire used in this study consisted of two main parts. In Part 1, the participants were asked to confirm if they were pharmacists practicing in Macao and to provide their demographic information (such as gender, age, highest qualification, years of practice as pharmacists, and the major area of practice). In Part 2, there were five main sub-sections, each of which contained a set of statements that measured A (5 statements), SN (5 statements), PBC (5 statements), PB (10 statements), and the intention to practice (10 statements). Participants were asked to rate their level of agreement on these items using a 5-point Likert scale with possible answers being strongly disagree, disagree, neutral, agree, and strongly agree.

Following the TPB framework, the items about A, SN, and PBC in the initial instrument were generated based on previous studies which employed TPB to explore factors associated with the intention of pharmacists to perform other cognitive services [including clinical pharmacy services (38), collaborative intervention (45), and medication management services (30)] and explored the role of pharmacists in public health (46, 47). The items about COVID-19 related duties to measure PB and the intention of the participants were based on an integrative analysis of the FIP-guiding document “COVID-19: Guidelines for Pharmacists and the Pharmacy Workforce” (6), and recent literature (7, 9, 42). The statements were designed and worded specifically to the context of the pharmacist profession in Macao. For instance, a growing discussion has been made about community pharmacists taking part in administering vaccinations as a key element of preventive services to improve access to flu vaccines and others for those who need them (48–50). However, in Macao, pharmacists by law are not allowed to perform any invasive procedure (including administering vaccines), so this was not considered as one of the COVID-19 duties in this study.

To ensure the theoretical constructs (A, SN, PBC, PC, and intention) were appropriately represented in the questionnaire, the initial instrument was first assessed by four researchers experienced in quantitative studies and pharmacy practice through a focus group. To ensure face validity of the questionnaire, they were also asked to evaluate if the statements in the questionnaire would allow reasonable and operational measurements of each TPB key variables and intention and to elaborate further on how to improve the validity of the questionnaire design. Based on their feedback, adjustments to the wordings to improve clarity were made. The revised instrument was then pilot tested on a convenience sample of six pharmacists from the community, hospital, or regulation sectors. They were requested to specifically evaluate whether the statements fully reflected what might constitute A and SN of pharmacists, the factors which might affect the ability of pharmacists to perform COVID-19 related duties (PBC), and the major components of COVID-19 duties for pharmacists in Macao (PB and intention). They all agreed that the questions were straightforward and easy to understand, confirming the face and content validity of the questionnaire. No removal of the original statements or addition of new ones was needed.

### Sampling

Although the present study focused on the COVID-19-related behavior of pharmacists in a community setting, pharmacists from non-community settings were also invited to participate, considering the mobilization of the pharmacist workforce to deal with public health emergencies being part of the management strategies. This study was to investigate the main factors affecting the intention-to-practice COVID-19-related duties of pharmacists in Macao, so it was important to collect responses from as many practicing pharmacists as possible. As such, invitations were sent or extended to all pharmacists practicing in Macao regardless of their sectors (*n* = 520). According to the sample size calculation by the online survey platform employed in this study, the valid sample size required for this study was determined at a minimum of 222 (confidence level 95%, a margin of error 5%).

### Data Collection

On one hand, publicly available information, such as the name of the registered pharmacists and the details of their workplace in Macao, was collected from the official website of the Health Bureau. An invitation letter that included a Participant Information Statement and a link to the online survey was sent to the pharmacists. On the other hand, the only two pharmacist professional organizations in Macao were invited to support this study. Upon their agreement, they helped distribute the survey invitations to their member pharmacists who collectively represented the majority of the pharmacist workforce in the region. The online questionnaire was hosted by the online questionnaire distribution company “Survey Monkey”. To ensure the completeness of the answers, the logic function requiring an answer to every question before submission available at the online survey platform was adopted. Likewise, to minimize the risks of double entries or duplication of entries by the pharmacists, the setting allowing only one attempt per device was employed. In the Participant Information Statement, it was also stated that, by submitting the survey, it was assumed that the participants were providing consent to take part in the study.

### Data Analysis

The survey responses were analyzed using the Statistical Package for Social Sciences (SPSS) version 24 software for Windows. The demographic data of the respondents were analyzed using descriptive statistics. In addition to descriptive statistics, Pearson's correlation was used to determine the strengths of the association between the intention and the four key variables. Since the instrument was self-developed, statistical analysis was conducted to determine the internal consistency reliability (Pearson's correlation coefficients, Cronbach's alpha coefficients and Composite Reliability Index among the sub-items of each construct), the convergent validity [factor loadings and average variance extracted (AVE)], and the discriminant validity (square roots of the AVE of each construct) among the constructs of the instrument. A multiple linear regression analysis was carried out on the data, with intention as the outcome factor and A, SN, PBC, and PB as the predictor variables. Whenever the *P*-value was found to be smaller than 0.05, the association would be considered statistically significant at a confidence level of 95%.

## Results

All the pharmacists practicing in Macau were invited to participate in this study, and 296 questionnaires were completed and valid for inclusion, giving a response rate of 56.9% (296/520). In other words, at least one in two pharmacists practicing in Macao participated in this study. After the online survey was closed, random follow-up contact with 20 pharmacists (*via* phone calls to randomly selected community pharmacies) found that the main reason for not participating was a lack of time.

The demographic information and relevant characteristics of the participants are presented in Supplementary Table 1. Among the 296 respondents, 165 (55.7%) were female and 221 (74.6%) were aged 26–40 years. Approximately one-fifth of the respondents (21.2%) had master's or doctoral degrees. Many respondents (65.5%) were junior pharmacists having less than 3 years of working experience. The majority of respondents (69.6%) were community pharmacists either at community pharmacies or other community settings, such as the public health centers. The demographic attributes of the participants were consistent with those of the pharmacist workforce in Macao in terms of age estimated by the year of registration and the registered workplace (43).

In Supplementary Table 2, the ratings of individual survey statements under each of the four key variables (A, SN, PBC, and PB) and the intention are presented together with the percentage of respondents giving positive (strongly agree/agree), neutral, and negative (strongly disagree/disagree) responses. Descriptive statistics, such as the mean and SD, of the ratings for each statement and the average mean and the percentage of positive, neutral, and negative responses for each TPB key variable and intention were also listed.

Overall, among the four TPB key variables, respondents rated the highest (average mean = 4.06 ± 0.52, % positive responses = 85.9%) for A toward their extended role in COVID-19 management, which was closely followed by PB (average mean = 4.03 ± 0.54, % positive responses = 84.9%). In contrast, the ratings for SN (average mean = 3.71 ± 0.58, % positive responses = 63.1%) and PBC (average mean = 3.76 ± 0.65, % positive responses = 67.6%) were lower.

In terms of the variable A, the respondents tended to agree that managing the COVID-19 infection outbreak was a valuable opportunity for the pharmacist profession to contribute to the community, even if this increased their workload. They were less likely to agree that their role was important to the successful management of the infection outbreak, and taking part in the COVID-19 management was important to move the pharmacist profession forward. Nevertheless, 85.9% of the respondents agreed that it was important for them to play a role in managing the COVID-19 infection.

In terms of the variable SN, the respondents were more likely to agree that the pharmacy personnel, whose opinions they valued, and the health care professionals would expect them to take part in managing the COVID-19 outbreak. They were less likely to agree that their patients, customers, and the community would have the same expectations. Only 51.4% of the respondents agreed that the people who were important to them would expect them to take part in managing the infection outbreak.

In terms of the variable PBC, the respondents were more likely to believe that they had the necessary knowledge and skills to take part in COVID-19 management with 83.8% of them giving positive responses. About 71.3% agreed that they had access to reliable information sources and guidelines relevant to COVID-19 infection. In contrast, the respondents were less likely to agree that they had sufficient hardware support, software support, or the time to perform COVID-19-related duties.

In terms of the variable PB, the frequency of involvement in COVID-19 management as reported by the respondents was generally high, which was indicated by the mean score of 4.03 for the 10 PB construct statements. They were most likely to agree that they had educated the community about personal and environmental hygiene, provided reassurance to the community to minimize panic, and ensured the continuity in the supply of medicines, face masks, and other related products. In contrast, they were less likely to agree that they had performed active surveillance of suspicious cases among the population and that they were prepared to make an appropriate referral of suspected patients according to the official protocol. While 84.9% of the respondents indicated that they had been involved in COVID-19 management, only 3.3% of the respondents indicated otherwise.

The respondents showed a stronger intention to practice for COVID-19 management. The mean score for the 10 statements was 4.19 with 91.9% of the respondents who agreed or strongly agreed that they intended to practice the COVID-19-related duties as listed. Similar to their experiences, the respondents rated the following COVID-19-related duties highest: educating the community about personal and environmental hygiene (mean score = 4.26 ± 0.58), providing assurance to the public to minimize panic (mean score = 4.26 ± 0.57), ensuring the continuity in the supply of medicine (mean score = 4.25 ± 0.63), face masks, and related products (mean score = 4.23 ± 0.63), and addressing the public inquiries with up-to-date information about the infection progress (mean score = 4.21 ± 0.59). Comparatively, the level of the intention of the respondents to perform active surveillance (mean score = 4.07 ± 0.69) and make referrals of suspected cases (mean score = 4.10 ± 0.65) was relatively low. Nevertheless, a total of 91.9% of the respondents agreed or strongly agreed that they intended to practice the COVID-19 duties.

Considering the survey tool was self-developed, the reliability and validity were evaluated. The Cronbach's α (R), AVE, composite reliability (CR), and the correlations among the variables are reported in Supplementary Table 3. The results suggest that the measures in the survey tool were of reasonable reliability and validity. First, all the scales showed good internal consistency reliability: the Cronbach's alpha (ranging between 0.82 and 0.94) and Composite Reliability Index (ranging between 0.83 and 1.18) of each construct exceeded the threshold of 0.7. Second, as shown in Supplementary Table 4, all the scales met the requirement of convergent validity as each item has a high and significant loading on its construct (>0.60) and the AVEs of all the constructs were above the threshold of 0.5 (ranging between 0.51 and 0.62). Third, the square root of AVE of each construct was greater than the cross-construct correlations, suggesting satisfactory discriminant validity.

As the hypotheses suggested, favorable A, positive SN, strong PBC, and frequent PB of pharmacists are the predictors of intention. In Model 1, as shown in Supplementary Table 5, the coefficients indicated that A (β = 0.25, *P* < 0.001) and PB (β = 0.62, *P* < 0.001) were significant predictors of intention, whereas SN (β = −0.01, *P* = 0.86) and PBC (β = 0.02, *P* = 0.59) were not significantly correlated with the intention. In other words, only hypotheses H1 [favorable attitude (A) is a positive and significant predictor of intention-to-practice COVID-19-related duties] and H4 [frequent past behaviors (PBs) related to COVID-19 is a positive and significant intention-to-practice COVID-19-related duties] are supported. A total of 72.5% (R = 0.851, adjusted R2 = 0.721.) of the variance in the intention-to-practice COVID-19-related duties can be explained by the two predictors A and PB (F = 191.626, d.f. = 4, *P* < 0.001). Between these two statistically significant predictors, the frequency of PBs related to COVID-19 management of the respondents had a stronger influence on their intention to practice (β = 0.66) as compared to that of A (β = 0.25).

Additional logistic regression was performed to evaluate the differences in the predictive efficacy of the TPB framework with or without the key variable of PB. In Model 2, only A, SN, and PBC were tested as possible predictors. Only 54.7% (R = 0.740, adjusted R2 = 0.345) of the variance in the intention to practice the COVID-19-related duties can be explained by these three constructs (F = 117.734, d.f. = 3, *P* < 0.001). The coefficients indicated that A (β = 0.42, *P* < 0.001), PBC (β = 0.28, *P* < 0.001), and SN (β = 0.18, *P* = 0.002) were all significant predictors of intention in the descending order. Comparing the two models, PB was found to contribute an extra 17.8% variance (72.5% variance explained by Model 1, 54.7% variance explained by Model 2) to the prediction of intention.

## Discussion

To our knowledge, this is the first known study that used the framework of the TPB to quantitatively examine the intention of pharmacists to contribute to COVID-19 management through expanding their role in public health. Over 90% of the participating pharmacists intended to extend their scope of practice to include COVID-19-related practice at the community setting. In general, they showed a positive A toward this extended role and many of them already had the practice experiences. However, fewer pharmacists believed that, while others considered it was important for pharmacists to do so or they were well supported for such undertaking. In the TPB model that used A, SN, PBC, and PB as possible predictors, 72.5% of the variance in intention to practice the COVID-19-related duties could be explained by two of the predictors with PB being a stronger predictor than A. Compared with the model which only used A, SN, and PBC as possible predictors, 54.7% variance of intention was explained, indicating an extra 17.8% variance of intention contributed by PB.

An important goal of this research was to identify the priority of actions needed to support the extended role of pharmacists in COVID-19 management for a more seamless integration of pharmacy practice to the public health network. As hypothesized, A, SN, PBC, and PB of pharmacists were significant predictors of the intention-to-undertake COVID-19 duties as the extended scope of practice in public health. In Model 1, which explained 72.5% of the variance in intention to carry out COVID-19-related duties, A and PB of pharmacists were significant predictors of intention, whereas SN and PBC were not (Model 1 in Supplementary Table 5). Nevertheless, in Model 2, which did not take into account PB but still effectively explained 54.7% of the variance in intention, A, PBC, and SN were all significant predictors of intention. It is to the interpretation of authors that, while PB was shown to be a distinctively significant predictor of the intention of the pharmacists followed by A, PBC and SN were also important variables worth exploring when deciding on an action plan that aimed to support pharmacists extending their role associated with the COVID-19 pandemic.

The sampled pharmacists in this study showed a strong intention to extend their role in public health to contribute to the management of the COVID-19 pandemic. Being among the community, they were well-positioned at the interface between the government and the public to carry out public health activities during infection outbreak (6). Psychologically, they were prepared to take on their role in managing the COVID-19 pandemic. Over 90% of the respondents considered their role to be an opportunity to contribute to the community, reflecting the public interests being their first priority. At the same time, most of them indicated that being able to participate in the management of COVID-19 was also important to them personally despite the increased workload, an attitude shared by the pharmacists in the United States who also believed that participating in public health activities related to emergency preparedness and response was important for the pharmacy profession (51). Similarly, pharmacists in Canada also recognized that times of crises were also new opportunities for them to evolve from their traditional roles and to apply existing knowledge and skills for new tasks (52).

Practically, many pharmacists in Macau had been mobilized to serve the community ever since the outbreak hit the city in January 2020 (42). This could explain why the majority of the respondents scored high in the rating for PB in this study. Just like many pharmacists elsewhere (7), they took up the responsibilities of the first contact point to the health system through informing and educating the general public about the outbreak, addressing their concerns and alleviating panic, promoting personal and environmental hygiene, being vigilant about suspected cases, and taking part in a coordinated effort led by the government. However, throughout the COVID-19 pandemic, pharmacists were rarely considered as essential health care human resources in the frontline (53). Their role and potential to contribute had been under-recognized at least from their perspectives. In this study, at least 40% of the pharmacists were not sure whether the public would expect them to play a role in COVID-19 and around 30% of them doubted whether other health care professionals would consider them part of the effort against COVID-19. It is anticipated that this study could raise awareness especially among the policymakers about the contributions that pharmacists may make to COVID-19 management through public health interventions.

The study results also indicated some challenges that pharmacists had to handle when practicing COVID-19 duties in a community setting. The physical constraints of limited space and information system, the lack of pharmacy staff, and the intense workload appeared to be the most concerning for pharmacists as reported in this study. In line with the experiences in European countries, Canada, and the United States, some of the most common difficulties were to ensure and promote social distancing and prevent overcrowding of the pharmacy (54). Understandably, this was particularly challenging due to an increased number of patients and customers especially at the beginning of the pandemic. Due to emotional stress or physical conditions, the workplace might be subject to the inevitable shortage of staff due to sick leaves or even unexpected quarantine (55). To assist behavioral changes of the pharmacists associated with providing public health services in community pharmacy, actions may be needed at the levels of government, general public, pharmacy management, and capacity building (11, 12, 19, 56, 57).

The significance of adopting the TPB model used in this study was at least 3-fold. First, the instrument developed for this study has been shown to have acceptable reliability and validity, and the results reaffirm that the TPB is a good framework in the pharmacy area for measuring the intention of pharmacists to undertake clinical services. Among the numerous examples, He et al. (38) found that the TPB model could help explain the intention of pharmacists to provide core clinical pharmacy services. In another study, the TPB was shown to have the utility in predicting theintention of pharmacists to report adverse drug event accounting (31). The findings from the present study are likely to help address previous concerns about the underutilization of theoretical models to explain the perceptions of pharmacists about providing professional services and the reporting quality of the psychometric properties, which may collectively improve the credibility of the research findings (28, 58).

Second, the predictive efficacy of the TPB model to explain the intention of pharmacists to undertake COVID-19-related duties at the community level in the present study was favorably high. In 2001, a meta-analytic review that involved 185 independent studies concluded that the TPB accounted for 39% of the variance in intention on average in general (27). In 2008, another review that involved 78 studies focusing on the routine clinical practice of health care professionals concluded that the TPB model was an appropriate theory to predict intention, which might explain 59% of the variance in intention. Other empirical studies concluded lower predictive usefulness of the TPB in pharmacy practice, showing that the TPB model could only explain 61% of the variance in the intention of pharmacists to perform systematic ADT monitoring and report on such monitoring (59), 54.0% of the variance in the intention of pharmacists to provide core clinical pharmacy practice (38), and 32% of the variance in the intention of pharmacists to report medication safety incidents (60). Such differences may be explained by the additional variables included in the TPB model design, which would be discussed further in the following.

The third key significance of the TPB model used in the present study was the inclusion of PB as an additional key variable. Logistic regression showed that the TPB model which took into account PB (72.5% explained intention) had much higher usefulness in predicting the intention of pharmacists than the TPB model without PB (54.7% explained intention). Indeed, the measurement of past behavior was the utmost predictor of the intention of pharmacists to practice COVID-19-related duties at the community level, as shown in the present study. Such findings supported the previous arguments about PB being the best predictor of future behavior (36, 61) and PB being able to add predictive value statistically (35). Nevertheless, the studies in the pharmacy sector that considered PB as a relevant variable in the TPB model are only limited (31, 38). However, it has been discussed that PB might be a significant predictor of intention only when the TPB cognitions were unstable (62, 63). Future studies should continue to investigate if and how PB may contribute to the predictability of the TPB in pharmacy practice.

## Limitations

This study had a number of limitations. Due to social desirability, self-reported measures of behavioral intention and PB in this study could be overrated and overestimated probably due to the association between PB and intention (38). Second, due to the nature of the cross-sectional study, no causal inferences could be made between the key TPB factors and the intention based on the findings of the present study. In addition, the associations identified in this study mostly involved psychosocial factors. External factors, such as rewards of action, motivation to comply, and policy support, which might have an impact on attitudes, subjective norms, and perceived behavioral control, respectively, and thus might further impact the intention of pharmacists, were not considered in this study. Future studies are warranted to explore environmental factors that were not accounted for in this study but may play a role in predicting the intention of pharmacists to undertake COVID-19-related practice within the framework of TPB. Moreover, due to legal restrictions in their scope of practice, the community pharmacy practice in Macao did not involve COVID-19 screening or tele-pharmacy and would not involve administering vaccination as would have been expected of pharmacists in the United States, the United Kingdom, and Canada (53). These limitations were reflected by the omission of the corresponding items in the questionnaire used in this study, compromising the generalizability of the study findings toward other pharmacist communities, which are subject to a different regulatory scheme.

## Conclusions

Pharmacists in Macau showed favorable intention to participate in COVID-19 management in the community setting. Although PB and A were significant and positive predictors of the intention of pharmacists, PBC and SN were also important factors. The results also suggested some challenges that pharmacists had to overcome when undertaking such an extended role. This study empirically tested and demonstrated the utility of the TPB to evaluate how psychosocial attributes and past behavior might guide behaviors related to the COVID-19 pandemic among pharmacists. Understanding the psychosocial factors that affect the engagement of pharmacists in COVID-19 management is essential to promote their role in the best interest of public health. There is a need for strategies to depict the value of the role of pharmacists to the public and healthcare professionals and optimize pharmacy management and other environmental enablers in order to foster the intention of pharmacists and hopefully facilitate the translation of intention into behavioral changes.

## Data Availability Statement

The original contributions presented in the study are included in the article/Supplementary Material, further inquiries can be directed to the corresponding author/s.

## Ethics Statement

The studies involving human participants were reviewed and approved by University of Macau. The patients/participants provided their written informed consent to participate in this study.

## Author Contributions

JL: carried out the literature review, collected and handled data, prepared data for analysis, performed statistics, interpreted the results, prepared the tables and figures, and drafted the manuscript. HH: interpreted the results and critically reviewed the manuscript. WL: assisted in data analysis and interpreted the results. CL: assisted in data analysis and reviewed the manuscript. CU: conceptualized and organized the study, carried out the literature review, performed statistics, interpreted the results, and critically reviewed and revised the manuscript. All authors contributed to the article and approved the submitted version.

## Conflict of Interest

The authors declare that the research was conducted in the absence of any commercial or financial relationships that could be construed as a potential conflict of interest.
