# Supplementary material for: Predicting Pharmacist Intention to Contribute to COVID-19 Management at the Community Level: A Cross-Sectional Survey Study
Source: Front Public Health. 2021 Jul 22;9:653335. doi: 10.3389/fpubh.2021.653335 (PMC8339551; doi:10.3389/fpubh.2021.653335)
Supplement: Supplementary file 1 [file Table_1.DOCX]

Supplementary Material

**Supplementary Material**

**Supplementary Table 1**

| **Supplementary Table 1: Demographics and relevant characteristics of participants (n=296)** | | | |
| --- | --- | --- | --- |
| Variables | Values | n | (%) |
| Gender | Male | 131 | 44.3% |
|  | Female | 165 | 55.7% |
| Age | <25 | 45 | 15.2% |
|  | 26 - 30 | 114 | 38.5% |
|  | 31 -  40 | 107 | 36.1% |
|  | 41 - 50 | 22 | 7.4% |
|  | 51 -  60 | 5 | 1.7% |
|  | 61 or over | 3 | 1.0% |
| Highest education level | Bachelor | 205 | 69.3% |
|  | Master | 60 | 20.3% |
|  | PhD | 3 | 1.0% |
|  | Others | 28 | 9.5% |
| Years of practice | <3 | 93 | 31.4% |
|  | 4-10 | 135 | 45.6% |
|  | 11-20 | 57 | 19.3% |
|  | >20 | 11 | 3.7% |
| Areas of practice | Community pharmacy | 194 | 65.5% |
|  | Health Care Center (public) | 12 | 4.1% |
|  | Hospital (public or private) | 31 | 10.5% |
|  | Elderly facility | 2 | 0.7% |
|  | Others | 57 | 19.3% |

**Supplementary Table 2**

| **Supplementary Table 2. Survey statements and descriptive statistics by each of the 4 constructs of the Theory of Planned Behavior (n=296)** | | | | | | | | | | |
| --- | --- | --- | --- | --- | --- | --- | --- | --- | --- | --- |
| **The 4 TPB constructs and I** | **Sub-items of each measure** | **Mean** | **Std. Deviation** | **Positive  (Strongly agree**  **or agree)** | | **Not sure** | | **Negative  (Strongly disagree/**  **disagree)** | |  |
|  |  |  |  | n | (%) | n | (%) | n | (%) |  |
| Attitude (A) | 1. It is important for me to play a role in managing infection outbreak. | 4.05 | +0.68 | 258 | 87.2% | 31 | 10.5% | 7 | 2.4% |  |
|  | 1. My role is important to the successful management of infection outbreak. | 4.01 | +0.66 | 246 | 83.1% | 45 | 15.2% | 5 | 1.7% |  |
|  | 1. Taking part in managing infection outbreak is a valuable opportunity for my role to contribute to the community. | 4.14 | +0.64 | 269 | 90.9% | 22 | 7.4% | 5 | 1.7% |  |
|  | 1. Taking part in managing infection outbreak is important in moving the profession of my role forward. | 4.04 | +0.71 | 246 | 83.1% | 43 | 14.5% | 7 | 2.4% |  |
|  | 1. I do not mind taking part in managing infection outbreak if that means an increase in my workload. | 4.09 | +0.69 | 252 | 85.1% | 39 | 13.2% | 5 | 1.7% |  |
|  | **Average** | **4.06** |  |  | **85.9%** |  | **12.2%** |  | **2.0%** |  |
| Subjective norm (SN) | 1. Most people who are important to me would expect me to take part in managing infection outbreak. | 3.52 | +0.81 | 152 | 51.4% | 120 | 40.5% | 24 | 8.1% |  |
|  | 1. Patients/customers would expect me to take part in managing infection outbreak. | 3.59 | +0.76 | 158 | 53.4% | 126 | 42.6% | 12 | 4.1% |  |
|  | 1. Healthcare professionals would expect me to take part in managing infection outbreak. | 3.80 | +0.69 | 209 | 70.6% | 80 | 27.0% | 7 | 2.4% |  |
|  | 1. Pharmacy personnel whose opinions I value would expect me to take part in managing infection outbreak. | 3.94 | +0.67 | 234 | 79.1% | 58 | 19.6% | 4 | 1.4% |  |
|  | 1. The community would expect me to take part in managing infection outbreak. | 3.71 | +0.70 | 181 | 61.1% | 111 | 37.5% | 4 | 1.4% |  |
|  | **Average** | **3.71** |  |  | **63.1%** |  | **33.4%** |  | **3.4%** |  |
| Perceived behavioral control (PBC) | 1. I have the necessary knowledge and skills. | 4.03 | +0.67 | 248 | 83.8% | 42 | 14.2% | 6 | 2.0% |  |
|  | 1. I have the necessary software support (e.g. sufficient staff). | 3.64 | +0.85 | 186 | 62.8% | 80 | 27.0% | 30 | 10.1% |  |
|  | 1. I have the sufficient hardware support (e.g. space, IT, etc). | 3.55 | +0.88 | 161 | 54.4% | 102 | 34.5% | 33 | 11.1% |  |
|  | 1. I have the necessary time. | 3.71 | +0.83 | 194 | 65.5% | 77 | 26.0% | 25 | 8.4% |  |
|  | 1. I have the access to reliable information sources and guidelines. | 3.85 | +0.79 | 211 | 71.3% | 73 | 24.7% | 12 | 4.1% |  |
|  | **Average** | **3.76** |  |  | **67.6%** |  | **25.3%** |  | **7.2%** |  |
| Past behaviors (PB) | 1. I have ensured the continuity of the supply of medicines. | 4.09 | +0.62 | 271 | 91.6% | 20 | 6.8% | 5 | 1.7% |  |
|  | 1. I have ensured the continuity of the supply of related products such as face masks, disinfectants, thermometers. | 4.06 | +0.75 | 249 | 84.1% | 35 | 11.8% | 12 | 4.1% |  |
|  | 1. I have ensured the continuity of patient care. | 4.02 | +0.66 | 253 | 85.5% | 36 | 12.2% | 7 | 2.4% |  |
|  | 1. I have addressed the public enquiries with accurate up-to-date information about the infection progress. | 4.06 | +0.66 | 259 | 87.5% | 29 | 9.8% | 8 | 2.7% |  |
|  | 1. I have provided reassurance to the community to minimize panic. | 4.14 | +0.70 | 260 | 87.8% | 28 | 9.5% | 8 | 2.7% |  |
|  | 1. I have performed active surveillance of suspicious cases among the population. | 3.81 | +0.84 | 209 | 70.6% | 67 | 22.6% | 20 | 6.8% |  |
|  | 1. I have been prepared to make referral of suspected patients according to the protocol developed by the health authority. | 3.92 | +0.75 | 230 | 77.7% | 54 | 18.2% | 12 | 4.1% |  |
|  | 1. I have educated the community about personal and environment hygiene. | 4.18 | +0.62 | 275 | 92.9% | 17 | 5.7% | 4 | 1.4% |  |
|  | 1. I have collaborated with other healthcare professionals in providing patient care and support. | 4.07 | +0.67 | 261 | 88.2% | 27 | 9.1% | 8 | 2.7% |  |
|  | 1. I have participated in the government strategies in managing the disease. | 4.00 | +0.79 | 245 | 82.8% | 37 | 12.5% | 14 | 4.7% |  |
|  | **Average** | **4.03** |  |  | **84.9%** |  | **11.8%** |  | **3.3%** |  |
| Intention to practice (I) | 1. I intent to ensure the continuity of the supply of essential medicines. | 4.25 | +0.63 | 280 | 94.6% | 13 | 4.4% | 3 | 1.0% |  |
|  | 1. I intent to ensure the continuity of the supply of related products such as masks, disinfectants, thermometers. | 4.23 | +0.63 | 270 | 91.2% | 24 | 8.1% | 2 | 0.7% |  |
|  | 1. I intent to ensure the continuity of patient care. | 4.16 | +0.65 | 267 | 90.2% | 24 | 8.1% | 5 | 1.7% |  |
|  | 1. I intent to address the public enquiries with accurate up-to-date information about the infection progress. | 4.21 | +0.59 | 280 | 94.6% | 14 | 4.7% | 2 | 0.7% |  |
|  | 1. I intent to provide reassurance to the community to minimize panic. | 4.26 | +0.57 | 282 | 95.3% | 13 | 4.4% | 1 | 0.3% |  |
|  | 1. I intent to perform active surveillance of suspicious cases among the population. | 4.07 | +0.69 | 254 | 85.8% | 36 | 12.2% | 6 | 2.0% |  |
|  | 1. I intent to become prepared to make referral of suspected patients according to the protocol developed by the health authority. | 4.10 | +0.65 | 263 | 88.9% | 29 | 9.8% | 4 | 1.4% |  |
|  | 1. Educating the community about personal and environment hygiene. | 4.26 | +0.58 | 282 | 95.3% | 12 | 4.1% | 2 | 0.7% |  |
|  | 1. I intent to collaborate with other healthcare professionals in providing patient care and support. | 4.19 | +0.59 | 274 | 92.6% | 20 | 6.8% | 2 | 0.7% |  |
|  | 1. I intent to participate in the government strategies in managing the disease. | 4.16 | +0.70 | 268 | 90.5% | 22 | 7.4% | 6 | 2.0% |  |
|  | **Average** | **4.19** |  |  | **91.9%** |  | **7.0%** |  | **1.1%** |  |

**Supplementary Table 3**

| **Supplementary Table 3. Correlations among the TPB constructs** | | | | | | | | | |  |
| --- | --- | --- | --- | --- | --- | --- | --- | --- | --- | --- |
|  | **Number of sub-items** | **R** | **AVE** | **CR** | **A** | **SN** | **PBC** | **PB** | **I** | |
| A | 5 | 0.82 | 0.51 | 0.83 | **0.72** |  |  |  |  |  |
| SN | 5 | 0.86 | 0.56 | 0.86 | 0.69** | **0.75** |  |  |  |  |
| PBC | 5 | 0.87 | 0.57 | 1.18 | 0.46** | 0.51** | **0.76** |  |  |  |
| PB | 10 | 0.92 | 0.55 | 1.09 | 0.63** | 0.54** | 0.65** | **0.74** |  |  |
| I | 10 | 0.94 | 0.62 | 1.07 | 0.67** | 0.61** | 0.56** | 0.73** | **0.79** |  |
| A, attitudes; SN, subjective norm; PB, past behaviours; PBC, perceived behavioural control; I, intention to practice; R, Cronbach’s α coefficients; AVE,average variance extracted; CR, composite reliability.  Square roots of AVE are bolded.  **Correlation is significant at the 0.01 level (2-tailed) | | | | | | | | | |  |

**Supplementary Table 4**

| **Supplementary Table 4. Factor loadings** | | | | | | |
| --- | --- | --- | --- | --- | --- | --- |
| The 4 TPB constructs and I | Sub-items of each measure | A | SN | PBC | PB | I |
|  |  |  |  |  |  |  |
| A | A.1 | 0.76 |  |  |  |  |
|  | A.2 | 0.78 |  |  |  |  |
|  | A.3 | 0.70 |  |  |  |  |
|  | A.4 | 0.70 |  |  |  |  |
|  | A.5 | 0.64 |  |  |  |  |
| SN | SN.1 |  | 0.74 |  |  |  |
|  | SN.2 |  | 0.77 |  |  |  |
|  | SN.3 |  | 0.72 |  |  |  |
|  | SN.4 |  | 0.75 |  |  |  |
|  | SN.5 |  | 0.76 |  |  |  |
| PBC | PBC.1 |  |  | 0.70 |  |  |
|  | PBC.2 |  |  | 0.87 |  |  |
|  | PBC.3 |  |  | 0.85 |  |  |
|  | PBC.4 |  |  | 0.64 |  |  |
|  | PBC.5 |  |  | 0.70 |  |  |
| PB | PB.1 |  |  |  | 0.65 |  |
|  | PB.2 |  |  |  | 0.69 |  |
|  | PB.3 |  |  |  | 0.76 |  |
|  | PB.4 |  |  |  | 0.79 |  |
|  | PB.5 |  |  |  | 0.71 |  |
|  | PB.6 |  |  |  | 0.80 |  |
|  | PB.7 |  |  |  | 0.82 |  |
|  | PB.8 |  |  |  | 0.73 |  |
|  | PB.9 |  |  |  | 0.76 |  |
|  | PB.10 |  |  |  | 0.67 |  |
| I | I.1 |  |  |  |  | 0.67 |
|  | I.2 |  |  |  |  | 0.75 |
|  | I.3 |  |  |  |  | 0.79 |
|  | I.4 |  |  |  |  | 0.85 |
|  | I.5 |  |  |  |  | 0.80 |
|  | I.6 |  |  |  |  | 0.74 |
|  | I.7 |  |  |  |  | 0.77 |
|  | I.8 |  |  |  |  | 0.85 |
|  | I.9 |  |  |  |  | 0.88 |
|  | I.10 |  |  |  |  | 0.76 |
| A, attitudes; SN, subjective norm; PB, past behaviors; PBC, perceived behavioral control; I, intention to practice  A.1-A.5 – the 5 sub-items measuring respondents’ attitudes; SN.1-AN.5 – the 5 sub-items measuring respondents’ subjective norms; PBC.1-PBC.5 – the 5 sub-items measuring respondents’ perceived behavioral control; PB.1-PB.10 – the 10 sub-items measuring respondents’ frequency of past behaviors related to COVID-19 management; I.1-I.10 – the 10 sub-items measuring respondents’ to practice COVID-19 related duties. | | | | | | |

**Supplementary Table 5**

| Supplementary Table 5. Results of multiple regression analysis for the TPB constructs (n=296) | | | | | | |
| --- | --- | --- | --- | --- | --- | --- |
| Predictor variables | Unstandardized regression coefficients B | Standard error SE | Standardized regression coefficients β | t | P | Significant predictor  (Yes/No) |
| **Model 1 (Possible predictors: A, SN, PBC and PB)** | | | | | |  |
| (Constant) | 0.643 | 0.134 |  | 4.787 | 0.000 |  |
| Attitude 🡪 I | 0.245 | 0.044 | 0.250 | 5.597 | 0.000 | Yes |
| SN 🡪 I | -0.007 | 0.040 | -0.008 | -0.177 | 0.859 | No |
| PBC 🡪 I | 0.017 | 0.032 | 0.022 | 0.546 | 0.586 | No |
| PB 🡪 I | 0.622 | 0.045 | 0.663 | 13.697 | 0.000 | Yes |
| Dependent Variable: intention F = 191.626, d.f. = 4, P < 0.001, R = 0.851, R2 = 0.725, adjusted R2 = 0.721. | | | | | | |
| **Model 2 (Possible predictors: A, SN and PBC)** | | | | | |  |
| (Constant) | 1.120 | 0.166 |  | 6.741 | 0.000 |  |
| Attitude 🡪 I | 0.413 | 0.054 | 0.421 | 7.666 | 0.000 | Yes |
| SN 🡪 I | 0.156 | 0.049 | 0.178 | 3.147 | 0.002 | Yes |
| PBC 🡪 I | 0.217 | 0.036 | 0.279 | 5.995 | 0.000 | Yes |
| Dependent Variable: intention F = 117.734, d.f. = 3, P < 0.001, R = 0.740, R2 = 0.547, adjusted R2 = 0.345. | | | | | | |
